# Critical roles of a housekeeping sortase of probiotic *Bifidobacterium bifidum* in bacterium–host cell crosstalk

**DOI:** 10.1016/j.isci.2021.103363

**Published:** 2021-10-28

**Authors:** Eiji Ishikawa, Tetsuya Yamada, Kazuaki Yamaji, Masaki Serata, Daichi Fujii, Yoshinori Umesaki, Hirokazu Tsuji, Koji Nomoto, Masahiro Ito, Nobuhiko Okada, Masato Nagaoka, Atsushi Gomi

**Affiliations:** 1 Yakult Central Institute, 5-11 Izumi, Kunitachi-shi, Tokyo 186-8650, Japan; 2 Tokyo University of Agriculture, 1-1-1 Sakuragaoka, Setagaya-ku, Tokyo 156-8502, Japan; 3 Department of Microbiology, School of Pharmacy, Kitasato University, 5-9-1 Shirokane, Minato-ku, Tokyo 108-8641, Japan

**Keywords:** Microbiology, Microbiome, Evolutionary ecology

## Abstract

*Bifidobacterium bifidum* YIT 10347 (BF-1) is adhesive *in vitro*. Here we studied the molecular aspects of the BF-1 adhesion process. We identified and characterized non-adhesive mutants and found that a class E housekeeping sortase was critical for the adhesion to mucin. These mutants were significantly less adhesive to GCIY cells than was the wild type (WT), which protected GCIY cells against acid treatment more than did a non-adhesive mutant. The non-adhesive mutants aberrantly accumulated precursors of putative sortase-dependent proteins (SDPs). Recombinant SDPs bound to mucin. Disruption of the housekeeping sortase influenced expression of SDPs and pilus components. Mutants defective in a pilin or in an SDP showed the same adhesion properties as WT. Therefore, multiple SDPs and pili seem to work cooperatively to achieve adhesion, and the housekeeping sortase is responsible for cell wall anchoring of its substrates to ensure their proper biological function.

## Highlights

• Non-adhesive mutants of *B. bifidum* lack a functional housekeeping sortase
• A non-adhesive mutant is less protective for cultured cells against acid stress
• The housekeeping sortase is important for anchoring adhesion factors to cell wall
• *B. bifidum* has species-specific sortase-dependent proteins

## Graphical abstract

**Figure:**
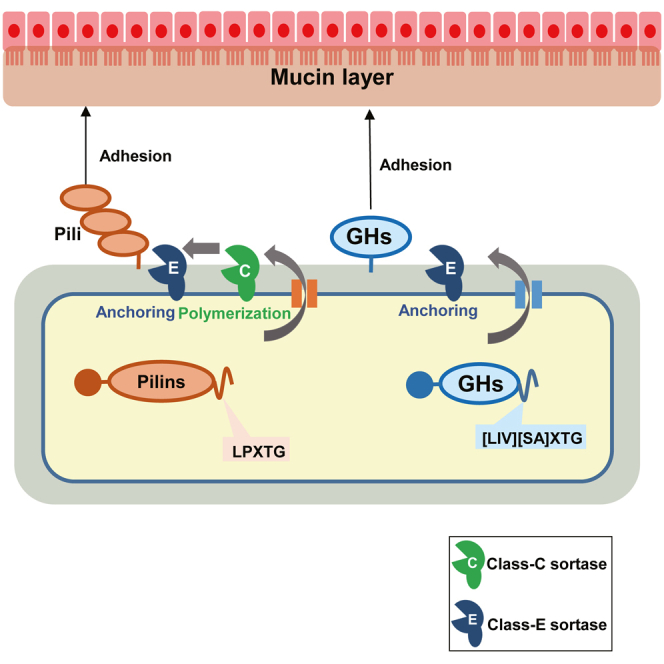

## Introduction

Bifidobacteria, a major bacterial group in the human large intestine, may provide human health benefits, but their numbers decrease upon weaning and aging (Mitsuoka, 1992; Woodmansey, 2007). Several bifidobacteria have been utilized as probiotics (Fuller, 1989), and *Bifidobacterium bifidum* is one of the major species used. Above all, *B. bifidum* has the unique ability to relieve symptoms of gastric disorders in humans (Miki et al., 2007; Gomi et al., 2015, 2018; Urita et al., 2015). The adhesion of *B. bifidum in vitro* is exceptionally strong among probiotics (Shibahara-Sone et al., 2016), and its mechanisms of adhesion and implications for host–bacteria crosstalk (i.e., probiotic effects) have interested many microbiologists (Serafini et al., 2013; Turroni et al., 2014).

Genome analysis has revealed that *B. bifidum* possesses unique genes responsible for mucin metabolism (Turroni et al., 2010); accordingly, mucin is used as a substrate in *B. bifidum*-specific selective culture media (Pechar et al., 2014). Several *B. bifidum* factors involved in adhesion to mucin or to cultured cells have been identified; these include sortase-dependent pili (Turroni et al., 2013), exo-α-sialidase (Nishiyama et al., 2017), BopA (Guglielmetti et al., 2008; Gleinser et al., 2012; Kainulainen et al., 2013), and transaldolase (Gonzalez-Rodriguez et al., 2012).

But reductionist approaches such as biochemistry or molecular biology occasionally encounter difficulties such as false positives, overlooking interactions among multiple factors, and failure to establish causal relationships. Consequently, the overall picture of the adhesion mechanism in *B. bifidum* remains obscure.

We therefore aimed to (i) develop a transposon mutagenesis system and ELISA-based adhesion assay system for *B. bifidum* YIT 10347 (BF-1) to enable a reverse-genetic approach, (ii) isolate non-adhesive BF-1 mutants, (iii) evaluate the effects of BF-1 adhesion on acid tolerance of cultured cells, and (iv) characterize the mechanism of BF-1 adhesion. These studies revealed that a housekeeping sortase is responsible for cell wall anchoring of its substrates to ensure their proper biological function. This is the first report to present conclusive information on the mechanism of adhesion or bacterium‒host cell interaction.

## Results

### Biochemical insights into mucin–BF-1 interaction

By modifying the ELISA technique, we developed a system to assay bacterial adhesion to mucin. The details of this system are described in STAR Methods. This system enabled us to investigate BF-1 adhesion to mucin immediately and precisely. To obtain biochemical insights into the adhesion of BF-1 to mucin, we evaluated the effects of protein denaturation on the adhesion. Heat-killed or proteinase K-treated BF-1 showed dramatically decreased mucin adhesion (Figure S1A), whereas antibody reactivity was not affected by these treatments (Figure S1B). Lectins such as wheat germ agglutinin (WGA) strongly inhibited BF-1 adhesion (Figure S1C), whereas soybean agglutinin (SBA) inhibited it slightly, suggesting that molecules involved in BF-1 adhesion are lectin-like proteins located on the bacterial cell surface and that ligands of mucin are sugar chains, to which WGA and/or SBA bind competitively.

### Development of transposon mutagenesis system for BF-1

After optimizing electroporation conditions (Figures S2A and S2B), we used a Tn5 transposon (Tn) mutagenesis system for *B. bifidum* (Figure S2C) and generated a disruptant gene library of 2,685 clones. Then, eight clones picked at random from the library were assessed through Southern hybridization to confirm that a single copy of Tn was inserted into each clone (Figure S2D). Furthermore, 100 randomly selected clones likewise contained single copies of Tn, which were inserted evenly throughout the genome (Figure S2E). The single-gene disruptant library comprising 2,685 clones was used for screening for non-adhesive mutants described below.

### High-throughput screening for non-adhesive mutants

We measured the growth of 2,685 clones and then tested them in the adhesion assay mentioned above. All clones were evaluated by plotting growth against adhesion (Figure S3A). Candidate non-adhesive mutants were further investigated with regard to their adhesion profile and antibody reactivity; some non-adhesive mutants, such as #1476 and #1543, showed normal antibody reactivity, whereas others, such as #1520, showed reduced antibody reactivity (Figures S3B and S3C). The insertion of Tn into BF1_0427 encoding a sortase was common to three non-adhesive mutants (#1476, #1543, and #1649) (Table 1). We consider BF1_0427 as a gene encoding a housekeeping sortase, because it is not clustered with genes encoding pilins (Spirig et al., 2011; Bradshaw et al., 2015) and is orthologous to BBPR_0099 in *B. bifidum* PRL2010.

**Table 1:** Screened mutants and transposon insertion loci

Strain	Insertion locus (bp)		Disrupted CDS	Ortholog in *B. bifidum* PRL2010	Predicted function	Mucin adhesion	Antibody reactivity
	Fwd	Rvs					
#679	1624671	1624679	BF1_1202	BBPR_1044	Hypothetical protein	+	+
#694	132478	132470	BF1_0110	BBPR_1591	hypothetical protein, DNA and RNA helicase-like protein	N.D.^a^	–
#697	1041395	1041403	BF1_0750	BBPR_0470	Aminoglycoside phosphotransferase precursor	+	+
#706	132478	132470	BF1_0110	BBPR_1591	Hypothetical protein, DNA and RNA helicase-like protein	N.D.	−
#959	133323	133331	BF1_0110	BBPR_1591	Hypothetical protein, DNA and RNA helicase-like protein	N.D.	−
#1242	134754	134762	BF1_0110	BBPR_1591	Hypothetical protein, DNA and RNA helicase-like protein	N.D.	−
#1476	590880	590872	BF1_0427	BBPR_0099	Sortase	−	+
#1520	1364150	1364158	BF1_0996	BBPR_0728	Hypothetical protein	N.D.	−
#1543	591215	591207	BF1_0427	BBPR_0099	Sortase	−	+
#1649	591226	591218	BF1_0427	BBPR_0099	Sortase	−	+
#2454	132242	132250	BF1_0110	BBPR_1591	Hypothetical protein, DNA and RNA helicase-like protein	N.D.	−
#2578	135902	135910	BF1_0111	BBPR_1592	Hypothetical protein, FHA-domain containing protein	+	+

a Not determined because of low antibody reactivity.

### Impact of bacterial adhesion on cultured cells

**Figure 1:**
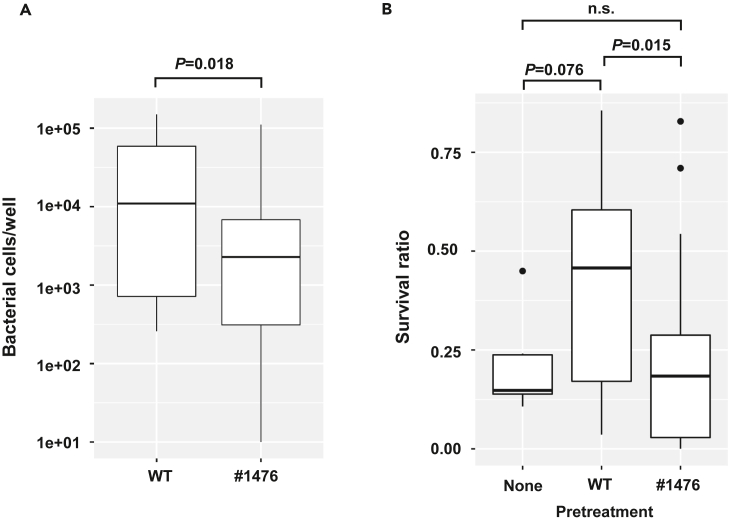
Bacterial adhesion properties affect the acid tolerance of cultured cells (A) Comparison of numbers of bacterial cells adhering to GCIY cells. The non-adhesive mutant #1476 showed significantly weaker adhesion than WT. Welch *t*-tests were performed on logarithmic values of bacterial counts. (B) GCIY cell survival after acid treatment. The survival ratio of GCIY cells pretreated with WT was significantly higher than that of cells pretreated with the non-adhesive mutant #1476. Tukey tests were performed on viable cell counts.

We then compared the adhesion properties of WT and the mutant #1476 by using GCIY cells, because #1476 was constitutively non-adhesive regardless of culture conditions, as described in the following section. This mutant showed significantly less adhesion to GCIY cells than did WT (Figure 1A), consistent with the results of the mucin adhesion assay.

We also evaluated the effects of BF-1 adhesion on the acid tolerance of GCIY cells. The survival ratio of acid-treated GCIY cells was significantly higher when pretreated with WT than with #1476 (Figure 1B), suggesting that the adhesion was critical to the protective effect.

### Characterization of non-adhesive mutants

The comparison of WT and #1476 in bacterial cell–GCIY cell co-incubation (Figure 1) suggested that the housekeeping sortase is involved in the adhesion, which is critical to bacteria–host cell interaction. Next, we identified the molecules involved in adhesion by comparing the non-adhesive mutants (#1476, #1543, and #1649) with WT.

**Figure 2:**
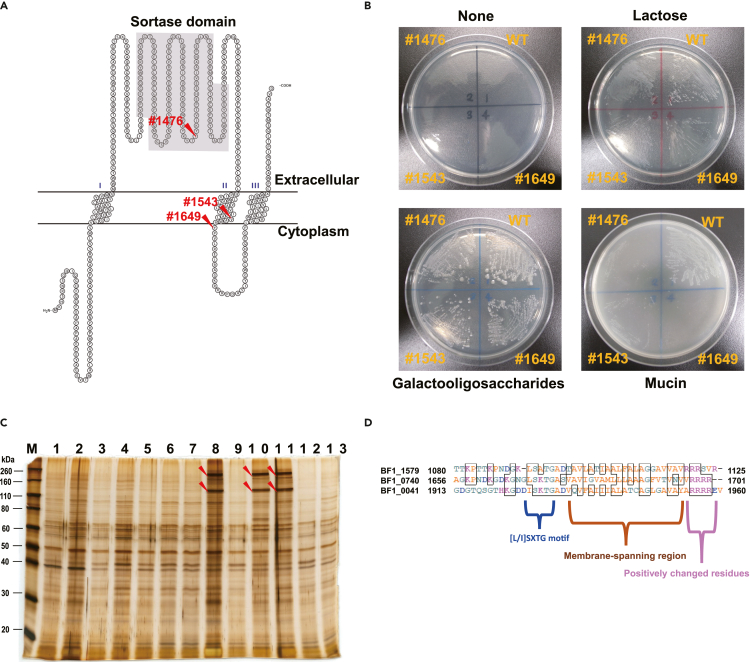
Characterization of the non-adhesive mutants (A) Predicted structure of the housekeeping sortase critical to mucin adhesion. The structure was predicted using SOSUI (https://harrier.nagahama-i-bio.ac.jp/sosui/). In each of the three independently isolated non-adhesive mutants (#1476, #1543, and #1649), the transposon was inserted into the gene encoding sortase. The extracellular sortase domain was disrupted in #1476, the second membrane-spanning region in #1543, and the cytosolic loop in #1649. (B) Effect of housekeeping sortase deficiency on substrate utilization. None of these mutants could grow on mucin. (C) SDS-PAGE of cell membrane proteins extracted from detected mutants. Three sortase-disrupted mutants (#1476, lane 8; #1543, lane 10; and #1649, lane 11) had several aberrantly accumulated proteins (arrowheads). Lane 1, WT; lane 2, #679; lane 3, #694; lane 4, #697; lane 5, #706; lane 6, #959; lane 7, #1242; lane 8, #1476; lane 9, #1520; lane 10, #1543; lane 11, #1649; lane 12, #2454; lane 13, #2578. (D) Sortase-dependent-protein (SDP)-like motifs identified in the C-termini of proteins that accumulated specifically in the mutants with disrupted housekeeping sortase.

We predicted the conformation of the housekeeping sortase, the disruption of which was common to all three of the non-adhesive mutants. This sortase appeared to be a transmembrane protein possessing three membrane-spanning regions (Figure 2A). The Tn was inserted into the sortase domain in #1476, into the second membrane-spanning region in #1543, and into the cytosolic loop in #1649. Mutants #1543 and #1649 partially recovered their adhesion when cultured in m-ILS medium. In contrast, #1476 was non-adhesive in both m-ILS and MRS media (Figure S4). These results suggest that the site of Tn insertion affected the adhesion property in different culture media, although the underlying mechanism remains unclear. None of the non-adhesive mutants could grow on mucin (Figure 2B), indicating that the housekeeping sortase is responsible for anchoring and processing of proteins that have a role to play in adhesion or in mucin degradation.

Cell membrane proteins extracted from the mutants listed in Table 1 were subjected to SDS-PAGE. Aberrantly accumulated proteins ranging 110–260 kDa were detected specifically in the three non-adhesive mutants (Figure 2C). Three proteins, BF1_0041 (BBPR_1514, β-N-acetylglucosaminidase), BF1_0740 (BBPR_0460, polysaccharide-degrading enzyme), and BF1_1579 (BBPR_1438, lacto-N-biosidase), were identified by proteomic analysis of these bands. Each of them had an N-terminal signal peptide and a C-terminal [L/I]SXTG motif followed by a membrane-spanning region and additional positively charged residues (Figure 2D).

### Sortase complementation of a non-adhesive mutant

**Figure 3:**
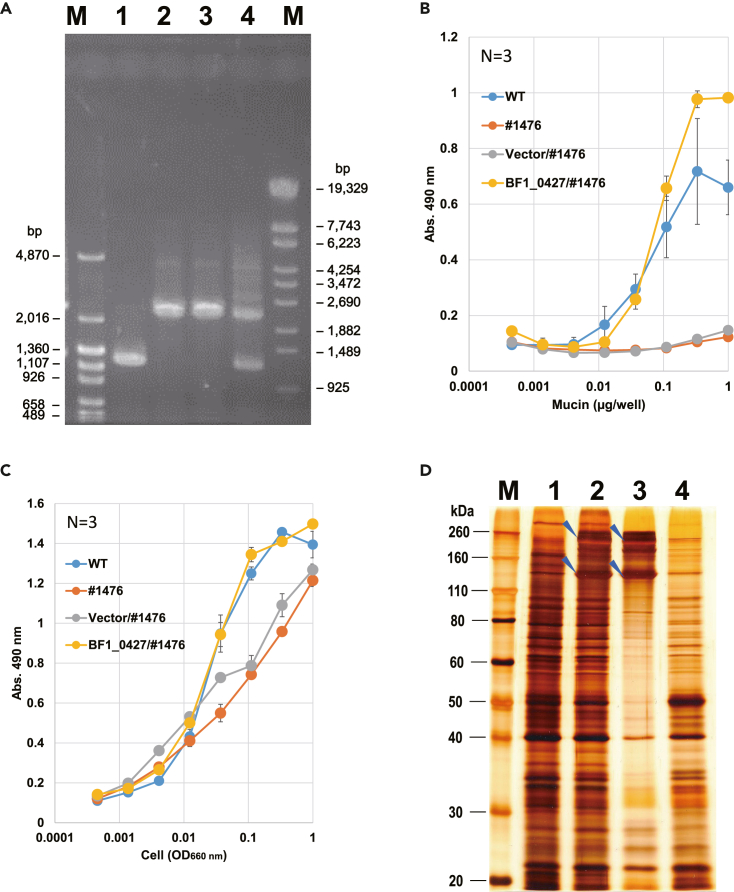
Genetic complementation of the housekeeping sortase (A) Confirmation of the complemented strain by PCR. Lane 1, WT; lane 2, #1476; lane 3, vector control of #1476; lane 4, complemented strain. *Bifidobacterium breve tuf* promoter-containing sequence and sortase gene were subcloned into a multi-cloning site of a shuttle vector (pBDSNBb1F), and the construct was used to transform #1476. The intact housekeeping sortase gene (approx. 1.2 kbp) was amplified in WT, whereas the gene with the inserted Tn (approx. 2.1 kbp) was amplified in both #1476 and the vector control. Both bands were detected in the complemented strain. (B) Mucin adhesion of the complemented strain. Bacteria adhering to mucin-coated plates were detected by ELISA as absorbance at 490 nm. (C) Antibody reactivity of the complemented strain. Serial dilutions of bacteria immobilized onto plates were detected by ELISA. Mucin adhesion recovered upon housekeeping sortase complementation, whereas antibody reactivity did not differ considerably among the clones. Data shown in (B) and (C) are means of 3 replicates ±SD. (D) SDS-PAGE of cell membrane proteins extracted from the complemented strain. Lane 1, WT; lane 2, #1476; lane 3, vector control of #1476; lane 4, complemented strain. Aberrantly accumulated proteins (arrowheads) disappeared upon complementation with the housekeeping sortase, consistent with the recovery of mucin adhesion.

The *B. breve tuf* promoter-containing sequence and BF1_0427 were subcloned into an *E. coli*–*Bifidobacterium* shuttle vector to yield a complementation plasmid for the housekeeping sortase. We used this construct to produce a complemented strain from #1476 (Figure 3A). Adhesion was recovered upon complementation (Figure 3B); antibody reactivity was almost the same among WT, #1476, the vector control, and the complemented strain (Figure 3C). Aberrantly accumulated proteins disappeared upon complementation (Figure 3D).

### Genome survey for putative sortase-dependent proteins

We surveyed the BF-1 genome and found several similar proteins but possessing a VAXTG motif instead of the [L/I]SXTG motif. Thus, we extensively searched for proteins having the signal peptide at the N-terminus and an [L/I/V][S/A]XTG motif, a membrane-spanning region, and positively charged residues at the C-terminus. We found 26 such sortase-dependent proteins (SDPs), most of which were glycoside hydrolases (GHs) (Table 2). These proteins were also found in *B. bifidum* PRL2010. Molecular weights of these proteins were entirely within the range of 110–260 kDa, being in good agreement with those estimated from the results of SDS-PAGE (Figures 2C and 3D).

**Table 2:** Putative sortase-dependent proteins

ID	Total AA	MW^a^	Predicted function	Signal peptide	[L/I/V][S/A]XTG motif^b^	C-terminal positive charge	Ortholog in *B. bifidum* PRL2010
BF1_0030	1,957	212,137	α-N-Acetylglucosaminidase	MMVSSARHRRGHSPEHPPTCHRSWRRRATALLVAMA	L^1925^SKTG	RSRRR	BBPR_1503
BF1_0032	1,597	167,780	Metallophosphoesterase	MRISTRIQSLLVSAALLVPLVATPVTA	L^1562^SQTG	RTSKRADRIR	BBPR_1505
BF1_0041	1,960	210,407	β-N-Acetylglucosaminidase	MRSKALGGLLAAALSLSPAVAIG	I^1926^SKTG	RRRREV	BBPR_1514
BF1_0056	1606	174333.12	β-N-Hexosaminidase	MSLTGVTAAQASDDNLALNQTVTASSYEVATTA	V^1576^AKTG	RKRRI	BBPR_nagZ2
BF1_0115	1,722	186,027	Sialidase	MMLATVMGPHFAGMRAQA	L^1694^SATG	AALALAKR	BBPR_1596
BF1_0151	1,139	123,735	β-N-Acetylglucosaminidase	MRANGNSTHEILGKIVTAIASIAMTAAFA	L^1107^SNTG	RKRIG	BBPR_1635
BF1_0172	739	78,939	Hypothetical protein	MALALVASLVFAAMPAA	L^706^SHTG	RRRSRR	BBPR_1657
BF1_0251	1338	138218.53	Minor extracellular serine protease	MAGSLPGTALAAPAQGDAAST	V^1303^AKTG	RRRDAVRR	BBPR_1740
BF1_0298	1,795	189,176	Sialidase	MTTIFRRATAKTLMRKLSGLLVAIAMLAVLPAGTISANA	V^1762^AKTG	RRRANR	BBPR_1793
BF1_0299	834	86,744	Exo-α-sialidase	MVRSTKPSLLRRLGALVAAAAMLVVLPAGVSTASAA	L^801^SKTG	RRRSAH	BBPR_1794
BF1_0365	1,220	129,346	α-L-Arabinofuranosidase	MARGWHRAGSHRFS	L^1188^SHTG	RRKRS	BBPR_0025
BF1_0485	561	58304.61	Sucrose symporter scrT	MTDDQQQPNEFPAPKPLPGSVYI	V^532^AVTG	KRKSNR	BBPR_scT
BF1_0505	297	31,431	Bacillolysin/Chitinase	VGEQHADGKCGLRVVRHG	L^256^SKTG	ASRGR	BBPR_0186
BF1_0510	1949	204449.73	α-Fucosidase	MPLVASCATVGMLLAGLPASAVA	V^1916^AKTG	RRKHSA	BBPR_0193
BF1_0575	1,714	184,068	Endo-α-N-acetylgalactosaminidase	MTALTDKLAMKAGSYSGT	I^1682^SKTG	RKRAE	BBPR_0264
BF1_0619	636	66,885	Hypothetical protein	MSNTNHSLTRGSIAAKA	L^586^SRTG	RPTSAR	BBPR_0323
BF1_0708	1,975	206,340	Autotransporter adhesin	MRRLVSPDAHRWAMPVIALVMLIGIIAGA	I^1916^SVTG	RIRRRSFGSM PSISTLASNRP	BBPR_0426
BF1_0740	1,701	178,321	Polysaccharide-degrading enzyme	MGTAFALIAPSSALA	L^1669^SKTG	RRRR	BBPR_0460
BF1_0762	1,935	206,662	β-Galactosidase	MAVRRLGGRIVAFA	L^1903^SKTG	RRKRS	BBPR_lacZ
BF1_1178	1060	113249.98	β-N-Acetylhexosaminidase	MNIKRRGLARFMSLICASAMLLVPASSALA	V^1028^AETG	RRQRR	BBPR_nagZ1
BF1_1357	956	100,401	Membrane-associated phospholipid phosphatase	MEAERMHKTMRKSAVLKGAVAGIASIAMLMSVSVTANAA	L^923^SKTG	RRKHAI	BBPR_1210
BF1_1442	829	88647.09	Lipoprotein	MNKRIAAVTATACMLFASVMIPAVSA	L^794^AHTG	RRLRISRE	BBPR_1292
BF1_1449	1280	134516.3	Glycosyl hydrolase	MCLAPLFSTNTAQA	I^1248^AATG	RKRRES	BBPR_1300
BF1_1506	1487	158087.4	α-1,3/4-Fucosidase	MLHTASRGCSRSWLRRLTALIAVSALAFVALPNVAVA	I^1454^AKTG	KRKSNR	BBPR_1360
BF1_1526	804	86460.1	Cation-transporting ATPase, E1-E2 family	MTGLTTSEVEERRARGEGETGARSVTKSTG	V^768^AMTG	RSRR	BBPR_ctpE
BF1_1579	1,125	121,024	Lacto-N-biosidase	MKLSCHNRNKRIKEVSMEKSSNRRFGVRTVAAIVAG	L^1092^SATG	RRRSVR	BBPR_1438

a Predicted molecular weight before processing by the signal peptidase and the housekeeping sortase.

b Superscripts represent the amino acid number from the N-terminus.

### Biophysical interaction between mucin and SDPs

To evaluate the contribution of SDPs to mucin adhesion, we prepared recombinant SDPs and investigated the binding of each SDP to mucin by surface plasmon resonance (SPR) assay. Of the 26 SDPs identified, 5 formed inclusion bodies or showed no expression, so 21 recombinant SDPs were used.

**Figure 4:**
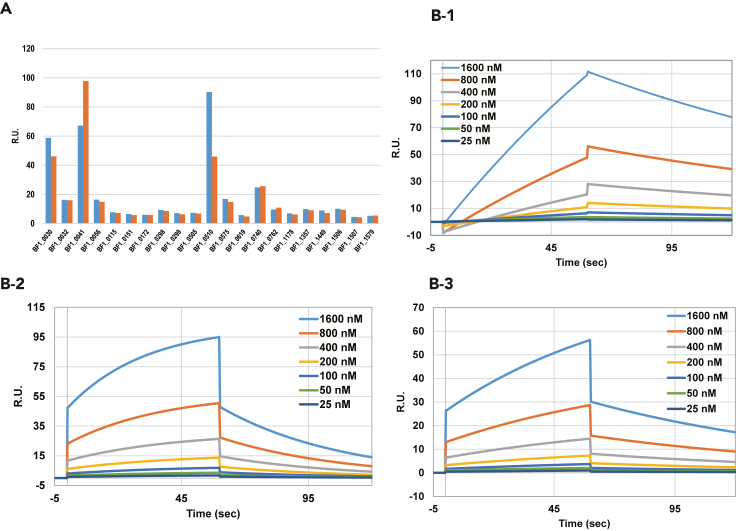
Interactions of putative sortase-dependent proteins with mucin Mucin was immobilized onto a sensor chip, and recombinant sortase-dependent proteins were subjected to surface plasmon resonance (SPR) assay. All proteins were screened for the maximum response (in resonance units, R.U.), and then the dose responses of adhesion candidates were investigated. The experiment was performed twice for each protein on different chips. (A) Maximum response of each sortase-dependent protein (100 nM). (B) SPR sensorgrams of interactions of BF1_0030 (panel B-1), BF1_0041 (panel B-2), and BF1_0056 (panel B-3) with mucin. Concentrations of the recombinant protein (25–1600 nM) are shown in each panel.

Next, we developed an SPR assay system to evaluate adhesion to mucin, using WGA and SBA as positive controls and bovine serum albumin as a negative control (Figure S5). In the SPR assay, each SDP appeared to have a unique affinity to mucin (Figure 4A). BF1_0030, BF1_0032, BF1_0041, BF1_0056, BF1_0510, BF1_0575, and BF1_0740 were selected as representative of SDPs for kinetic analysis. Of these, BF1_0030 (BBPR_1503), BF1_0041 (BBPR_1514), and BF1_0056 (BBPR_nagZ2) showed typical binding/dissociation curves and dose responses (Figure 4B), as did the lectins WGA and SBA, suggesting that multiple SDPs are involved in BF-1 adhesion to mucin.

### Effects of sortase disruption on BF-1 transcriptome

**Figure 5:**
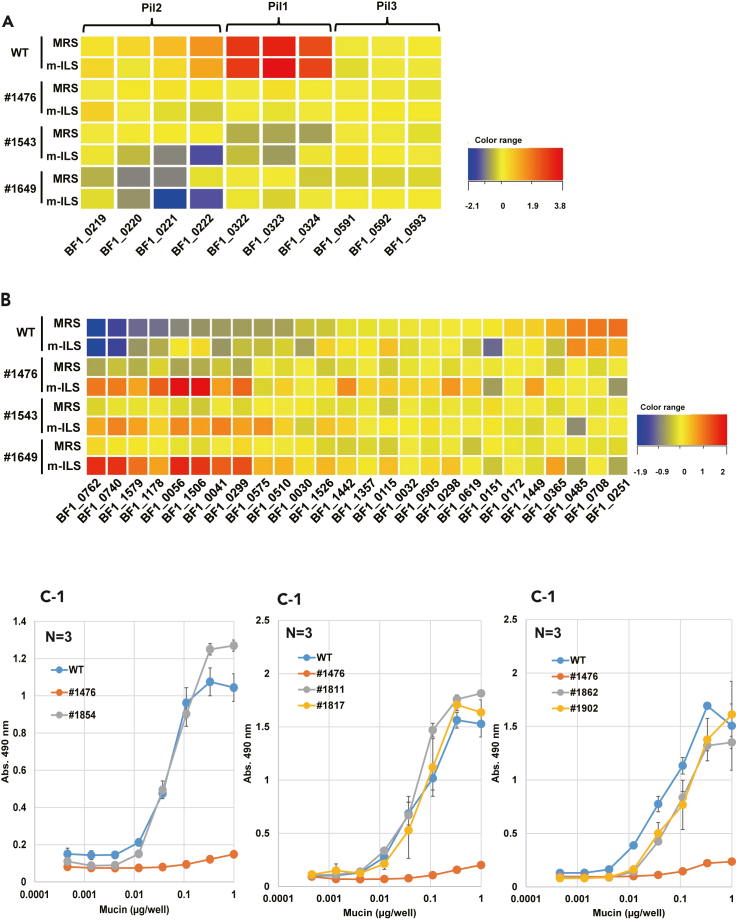
Effects of housekeeping sortase disruption on the expression of genes encoding pilins and putative SDPs, and adhesion properties of the pilin- or SDP-disrupted mutants (A) Heat map of three pilus operons. The operon comprising BF1_0322–BF1_0324 (Pil1 in *B. bifidum* PRL2010) was remarkably down-regulated in all three non-adhesive mutants, irrespective of medium composition. (B) Effects of housekeeping sortase disruption on SDP expression profiles. Aberrant proteins identified (BF1_0041, BF1_0740, and BF1_1579) were up-regulated in the non-adhesive mutants, especially when cultured in m-ILS medium. (C) Mucin adhesion properties of pilin- or SDP-disrupted mutants. Mucin adhesion of these mutants was almost the same as that of WT. #1854: ΔPil1 (BF1_0324); #1476: Δhousekeeping sortase (BF1_0427), negative control; #1811: Δexo-α-sialidase (BF1_0299); #1817: Δsialidase (BF1_0298); #1862: Δα-L-arabinofuranosidase (BF1_0365); #1902: Δβ-N-hexosaminidase (BF1_0056). Data shown in (C) are means of 3 replicates ± SD.

To explore the molecular mechanism of BF-1 adhesion, we compared transcriptomes among WT and non-adhesive mutants cultured in MRS or m-ILS medium (Figure S6). In all non-adhesive mutants regardless of culture medium, BF1_0322–0324 (Pil1 cluster [BBPR_1820–1822 in *B. bifidum* PRL2010]), BF1_1117–1120 (BBPR_937–940), and BF1_1439 (BBPR_1289) were down-regulated 5-fold, and BF1_0001 (BBPR_1472) was up-regulated 5-fold. Because pilins are regarded as adhesion factors (Turroni et al., 2013), we examined the effects of housekeeping sortase disruption on the expression of BF-1 pilins. Pil1 was highly expressed in WT but was remarkably down-regulated in the non-adhesive mutants. Pil2 (BBPR_1707–1709, no BF1_0221 in *B. bifidum* PRL2010) was moderately expressed in WT and was down-regulated approximately 2-fold in the non-adhesive mutants. Pil3 (BBPR_0282–0284) had relatively low expression, and its expression was not changed by housekeeping sortase deficiency (Figure 5A). These results indicate a strong interaction between the housekeeping sortase and pili, especially Pil1 and Pil2. BF-1 also has Type IVb tight adherence (Tad) pili (O'Connell et al., 2011), but the orthologous operon (BF1_0265–0271) was not influenced by housekeeping sortase disruption (data not shown).

Next, we evaluated the effects of housekeeping sortase disruption on SDP expression. SDPs with low expression in WT were up-regulated in all non-adhesive mutants, and such up-regulation was more remarkable in m-ILS medium than in MRS medium. Most of the up-regulated SDPs were GHs (Figure 5B, left). The aberrant proteins identified by proteomic analysis—BF1_0041, BF1_0740, and BF1_1579—were also remarkably up-regulated in the non-adhesive mutants. The expression of other SDPs was almost the same among the non-adhesive mutants and WT, or they were weakly down-regulated in the non-adhesive mutants. Most of these SDPs were not GHs (Figure 5B, right).

We suspected that the housekeeping sortase interacted with pili and/or SDPs, and we presumed that pili and/or SDPs were the adhesion factors; therefore, we examined the adhesion properties of pilin-disrupted or SDP-disrupted mutants. Because we had identified mutants with Tn inserted into pilin or SDP genes in our evaluation of the Tn mutagenesis system (Figure S2E), we examined these mutants in the mucin adhesion assay. Mutants #1854: ΔPil1 (BF1_0324), #1811: Δexo-α-sialidase (BF1_0299), #1817: Δsialidase (BF1_0298), #1862: Δα-L-arabinofuranosidase (BF1_0365), and #1902: Δβ-N-hexosaminidase (BF1_0056) showed the same adhesion properties as WT (Figures 5C1–5C3). These results suggest the presence of multiple adhesion factors and that a single-gene disruption does not critically affect BF-1 adhesion.

## Discussion

By using reverse genetics (i.e., the Tn mutagenesis system) and high-throughput screening (i.e., the ELISA-based mucin adhesion assay), we were able to isolate non-adhesive BF-1 mutants (Table 1). Adhesion of BF-1 and the mutants to mucin-producing GCIY cells was consistent with their adhesion to mucin; therefore, the main ligand would be mucin of GCIY cells. Adhesion was important for increasing acid tolerance of GCIY cells following BF-1 treatment (Figure 1B). Oral administration of *B. bifidum* OLB 6378 to rats activates Toll-like receptor-2 (TLR-2) to reduce apoptosis in the intestinal epithelium in necrotizing enterocolitis (Khailova et al., 2010), and cell-surface β-glucan/galactan of *B. bifidum* PRI1 induces regulatory T cells through a partially TLR-2-mediated mechanism (Verma et al., 2018). BF-1 exerts anti-ulcer effects via increasing the levels of epidermal growth factor and basic fibroblast growth factor *in* *vitro* and *in vivo* (Nagaoka et al., 1994). BF-1 also represses the expression of inflammatory cytokines induced by *Helicobacter pylori* infection via the NF-κB signaling pathway in GCIY cells (Shirasawa et al., 2010). Such effects might be related to the GCIY responses we observed.

Genes encoding class C sortases adjacent to genes encoding pilus components (pilins) have been studied in several bifidobacterial species (Milani et al., 2017). Genes encoding housekeeping sortases (class E) are not clustered with genes for their substrates. Housekeeping sortases appear to be involved in pilus attachment and/or formation of aerial hyphae in high-G + C bacterial species (Spirig et al., 2011; Bradshaw et al., 2015). As a result of the first-ever Tn mutagenesis system for *B. bifidum* that we originally developed, we were able to examine all the clones positively selected by antibiotic resistance. Consequently, 2,685 clones could be examined to discover the critical role of the housekeeping sortase in adhesion to mucin and/or to cultured cells.

Mucin adhesion was closely linked to mucin utilization (Figure 2B), suggesting that activation of SDPs involved in mucin degradation would require processing by the housekeeping sortase, anchoring to the cell wall, or both. Mucin adhesion of #1543 and #1649 changed depending on culture medium (Figure S4). Several SDPs may be induced by lactose, the main substrate in m-ILS (but not MRS, which contains glucose), and partially processed in #1543 or #1649, in which the housekeeping sortase domain was intact (Figure 2A).

Our results suggest that SDPs and pili cooperatively confer the bacterial adhesion property and compensate for one another. Indeed, most of the SDPs had lectin-like domains, bacterial IgG-like domains, and carbohydrate-binding domains (data not shown). In addition, pilin-disrupted or SDP-disrupted mutants would likely be present in the Tn-disruptant library (>2,500 clones), but none of the identified pilin-disrupted or SDP-disrupted mutants were non-adhesive. These circumstantial lines of evidence indirectly support the assumption that a single SDP or pilin disruption is not critical to BF-1 adhesion.

**Figure 6:**
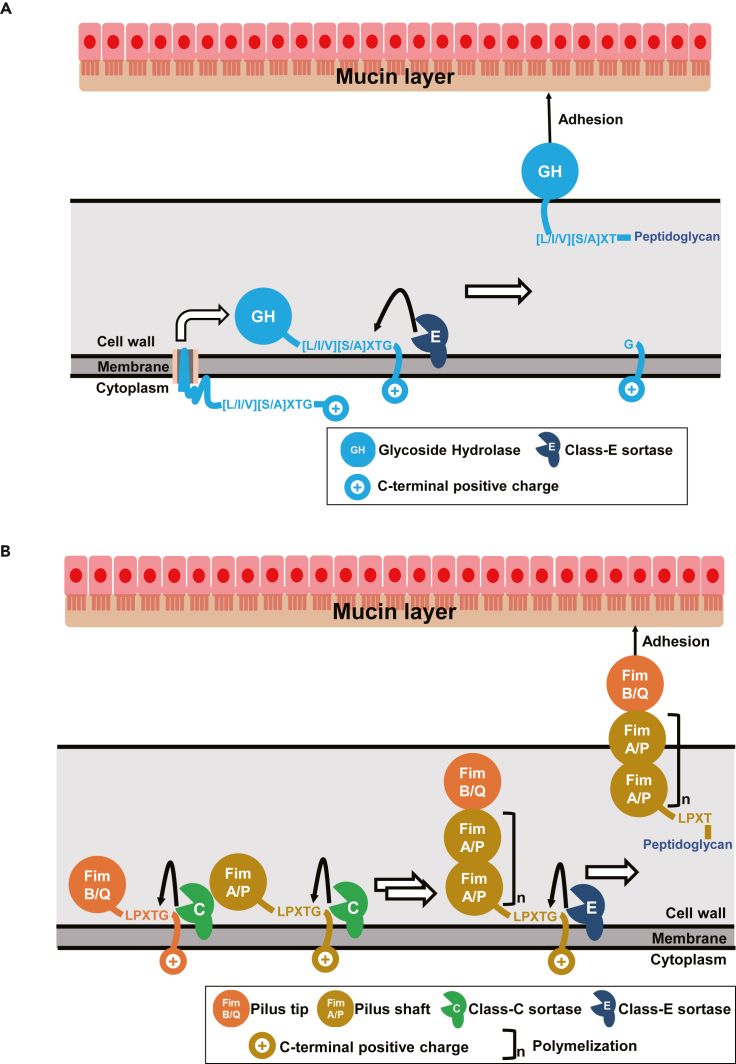
Model of the action of the housekeeping sortase in *B. bifidum* mucin adhesion (A) SDPs such as glycoside hydrolases (GHs) listed in Table 2 are anchored to the cell wall by the housekeeping class E sortase (BF1_0427). (B) Pilus components (Fim A/P and Fim B/Q) are polymerized by adjacent class C sortases (BF1_0222, BF1_0322, and BF1_0593), and the assembled precursors are anchored to the cell wall by the housekeeping class E sortase (BF1_0427). Pili and GHs are involved in mucin adhesion together.

Based on our results and previous reports (Turroni et al., 2013; Nishiyama et al., 2017), we propose a mechanism of BF-1 adhesion (Figure 6). SDPs with an [L/I/V][S/A]XTG motif, mainly GHs, are secreted, processed by the housekeeping class E sortase, and displayed on the cell wall. Consequently, these SDPs are involved in adhesion (Figure 6A). Pilus shaft components (Fim A/P) are polymerized and assembled with pilus tip components (Fim B/Q) by the adjacent class C sortases (Foroni et al., 2011). The produced pilus precursors are processed and attached to the cell surface by the housekeeping class E sortase. The displayed pili are involved in adhesion (Figure 6B). On the whole, SDPs and pili each exert adhesion activity, and the housekeeping sortase is a key enzyme in BF-1 adhesion.

Generally, housekeeping class E sortases recognize LAXTG as a sorting signal (Kattke et al., 2016), but BF1_0427 presumably recognizes [L/I/V][S/A/P]XTG, which deviates from motifs recognized by known class E sortases. Further structural studies will be necessary to reveal the usage of these unique sorting signals.

We searched the typical genomes of 17 bifidobacterial species (Schell et al., 2002; Sela et al., 2008; Ventura et al., 2009; Yasui et al., 2009; Turroni et al., 2010; O'Connell et al., 2011; Jans et al., 2013; Milani et al., 2013, 2014; Chen et al., 2015; Morita et al., 2015a, 2015b, 2015c, 2015d; Toh et al., 2015; Vazquez-Gutierrez et al., 2015) for orthologs of housekeeping sortase or SDPs, and found that BF1_0427 was conserved among all these species, whereas the SDPs, especially GHs (Table 2), were specific to *B. bifidum*. GHs identified and biochemically characterized in *B. bifidum* so far include BF1_0298 (Ashida et al., 2018), BF1_1449 (Wakinaka et al., 2013), BF1_0299 (Kiyohara et al., 2011), BF1_1506 (Ashida et al., 2009), BF1_1579 (Wada et al., 2008), BF1_0510 (Katayama et al., 2004, 2005), and BF1_0030 (Shimada et al., 2015). Some of these have been introduced into other bifidobacteria and have been shown to be functionally expressed in the bifidobacteria thus transformed (Ashida et al., 2009; Kiyohara et al., 2011). The housekeeping sortase, which is conserved among bifidobacteria, would be compatible with these SDPs, so the introduced SDP would be processed by the endogenous housekeeping sortase and be promptly attached onto the cell wall.

*Bifidobacterium bifidum* is unique among bifidobacterial species. For instance, by being able to utilize human milk oligosaccharides, *B. bifidum* is one of the major bacterial species among the first colonizers of the newborn's gastrointestinal tract (Duranti et al., 2019), and by being the only bifidobacterial species able to assimilate mucin (Pechar et al., 2014), it contributes to cross-feeding of other bacteria including bifidobacterial species (Egan et al., 2014; Turroni et al., 2015; Bunesova et al., 2018; Gotoh et al., 2018; Nishiyama et al., 2018; O'Connell et al., 2018; Centanni et al., 2019). Such characteristics might arise from the SDPs specific to *B. bifidum* (i.e., GHs). The reason why *B. bifidum* has developed such SDPs in evolution (e.g., adaptation to the environment of the gastrointestinal tract) remains unclear. Studies into why this is so will provide good opportunities to integrate microbial ecology and pan-genomics. The housekeeping sortase and SDPs would provide a new approach to studies on bifidobacteria.

In conclusion, the housekeeping sortase in *B. bifidum* is a key molecule both in adhesion to mucus and in bacterium–host cell interaction.

### Limitations of the study

Because BF-1 is adhesive to the gastric mucus *in vivo* (Shibahara-Sone et al., 2016), we used reagent-grade porcine stomach mucin for the large-scale screening. Yet, the bifidobacterial habitat is the large intestine; therefore, it might be better to check the adhesion properties of non-adhesive mutants using mucins purified from the human colon or a human colon cell line. The adhesion mechanism presented in this paper is based on *in vitro* study. As the next step, the adhesion mechanism *in vivo* could be investigated using animal models, including gnotobiotic mice or rats.

Microarray analysis revealed that housekeeping sortase would functionally interact with pili, but the other effects of sortase disruption remain obscure. Identification of other genes up-regulated or down-regulated in the non-adhesive mutants might provide insights into the pleiotropic effects of housekeeping sortase disruption. Although multiple SDPs or pili are assumed to be involved in BF-1 adhesion, the contribution of each SDP or pilus remains unclear, because their levels and adhesive activity have not been characterized in detail. An SDP-deletion or pilus-deletion mutant generated by genome editing would be useful for investigating the adhesion system.

Our next challenge would be to investigate the functional interactions of the sortase domain with the sorting signals (i.e., [L/I/V][S/A/P]XTG) *in vitro*. The reactions of the housekeeping sortase are carried out on the cell membrane, so that it might be difficult to recreate them on SPR chips. The co-crystal structures of the housekeeping sortase and the sorting signals would be helpful for investigating the functional interactions.

## STAR★Methods

### Key resources table

**Table:** 

REAGENT or RESOURCE	SOURCE	IDENTIFIER
**Antibodies**		

Horseradish peroxidase (HRP)-conjugated goat anti-rabbit Ig	SouthernBiotech	Cat#4010-05; RRID: AB_2632593
Rabbit anti-BF-1 custom polyclonal antibody	Eurofins	N/A

**Bacterial and virus strains**		

*Bifidobacterium bifidum* YIT 10347	NITE	FERM P-20569
*Escherichia coli* JM109	Takara Bio	Cat#9052

**Biological samples**		

Mucin from porcine stomach	Sigma-Aldrich	Cat#M1778
BigDye Terminator v3.1 Cycle Sequencing Kit	Thermo Fisher Scientific	Cat#4337455
Bovine Serum Albumin	Sigma-Aldrich	Cat#A9418
The BigDye XTerminator Purification Kit	Thermo Fisher Scientific	Cat#4376486
Mutanolysin	Sigma-Aldrich	Cat#M9901
QIAquick Gel Extraction Kit	Qiagen	Cat#28704
RNA Protect Bacteria Reagent	Qiagen	Cat#76506
Illustra Bacteria Genomicprep Mini Spin Kit	GE Healthcare Life Sciences	Cat#28904258
Lysozyme	Sigma-Aldrich	Cat#L6876
ProteoExtract All-in-One Trypsin Digestion Kit	Merck-Millipore	Cat#650212
Wheat Germ Agglutinin	Vector Labs	Cat#L-1020
RiboPure-Bacteria	Thermo Fisher Scientific	Cat#AM1925
Ni-NTA Spin Kit	Qiagen	Cat#31314
Soy Bean Agglutinin	Vector Labs	Cat#L-1010
AlkPhos Direct Labelling Module	GE Healthcare Life Sciences	Cat#RPN3680
CDP-Star Detection reagent	GE Healthcare Life Sciences	Cat#RPN3682

**Chemicals, peptides, and recombinant proteins**		

MRS medium	Difco Laboratories	Cat#DF0881-17-5
Eagle’s minimal essential medium	Nissui	Cat#5900
Luria–Bertani medium	Difco Laboratories	Cat#DF0446-07-5

**Deposited data**		

Complete genome sequence of *Bifidobacterium bifidum* YIT 10347	DDBJ/EMBL/GenBank	Accession#AP024712
Miroarray data of *Bifidobacteriumbifidum* YIT 10347 or non-adhesive mutants	GEO	Accession#GSE175843

**Experimental models: Cell lines**		

GCIY	RIKEN Cell Bank	Cat#RCB0555

**Oligonucleotides**		

	See Table S1 for a list of oligonucleotides used for preparing recombinant proteins	

**Recombinant DNA**		

EZ-Tn5 Custom Transposome Construction Kits	Epicentre	Cat#TNP10622
pUC19	Takara Bio	Cat#3219
In-Fusion HD Cloning Kit	Takara Bio	Cat#639648
pCold system	Takara Bio	Cat#3360

**Software and algorithms**		

GeneSpring 14	Agilent Technologies	N/A
R software	https://www.R-project.org/	N/A

### Resource availability

#### Lead contact

Further information and requests for resources and reagents should be directed to and will be fulfilled by the lead contact, Eiji Ishikawa (eiji-ishikawa@yakult.co.jp).

#### Materials availability

All materials are available from the corresponding author upon reasonable request.

### Experimental model and subject details

#### Bacterial strains and culture media

*Bifidobacterium bifidum* YIT 10347 (BF-1) was used as a wild-type (WT) strain. The complete genome sequence (2,149,912 bp) of BF-1 (GenBank: AP024712) was found to be similar to that of *B. bifidum* PRL2010 (Turroni et al., 2010). BF-1 and its mutants were anaerobically cultured at 37°C in de Man–Rogosa–Sharpe (MRS) medium (Difco Laboratories, Detroit, MI, USA) supplemented with 0.05% L-cysteine HCl or in modified ILS (m-ILS) medium containing 10 g trypticase peptone (Difco Laboratories), 5 g yeast extract (Difco Laboratories), 3 g tryptose (Difco Laboratories), 10 g lactose, 3 g KH_2_PO_4_, 3 g K_2_HPO_4_, 2 g tri-ammonium citrate, 1 mL pyruvate, 0.3 g L-cysteine HCl, 1 mL Tween 80 (Sigma-Aldrich, St. Louis, MO, USA), 0.575 g MgSO_4_·7H_2_0, 0.12 g MnSO_4_·4H_2_0, and 0.034 g FeSO_4_·7H_2_O in 1.0 L distilled water (pH 6.8).

To count viable bacteria (CFUs), serial dilutions of the samples were plated on agar plates containing MRS medium supplemented with 0.05% L-cysteine HCl and cultured at 37°C. Preparation of the dilutions and cultures was carried out anaerobically.

*Escherichia coli* JM109 (Takara Bio, Shiga, Japan) were used as competent cells for plasmid construction; they were aerobically cultured at 37°C in Luria–Bertani medium (Difco Laboratories).

#### Assay of bacterial adhesion to mucin

Mucin (porcine stomach mucin, Type III, bound sialic acid 0.5%–1.5%; Sigma-Aldrich) was serially diluted (0.0005‒1 μg/well) with 50 mM sodium carbonate buffer (pH 9.6) and immobilized in 96-well flat-bottom plates (Nunc MaxiSorp) at 4°C overnight. The immobilized mucin was blocked with PBS containing 1% gelatin (#1706537, Bio-Rad Laboratories). After removal of the blocking buffer, a bacterial suspension (OD_660 nm_ = 1.0) in PBS containing 1% gelatin was added to the plates and incubated at 37°C for 1 h, shaken at 1,200 rpm with a microplate shaker (Certomat® MT, B. Braun, Melsungen, Germany). After removal of the bacterial suspension, the plates with adhering bacteria were washed three times with PBS containing 0.1% Tween 20 (Bio-Rad Laboratories). Bacteria adhering to the immobilized mucin were measured by using a rabbit anti-BF-1 custom polyclonal antibody (Eurofins, Tokyo, Japan) as primary antibody and horseradish peroxidase (HRP)-conjugated goat anti-rabbit Ig (SouthernBiotech, Birmingham, AL, USA) as secondary antibody. After an OPD (*o*-phenylenediamine)-HRP reaction, the absorbance at 490 nm was measured using a plate reader (ARVO™ X3; PerkinElmer, Waltham, MA, USA). When evaluating the inhibitory effects of lectins (WGA and SBA; Vector Labs, Burlingame, CA, USA), the amount of mucin immobilized to each well was fixed at 1 μg/well, and immobilized mucin was pretreated with serially diluted lectins; thereafter, non-absorbed lectins were completely removed by aspiration before adding the bacterial suspension to avoid interactions between bacterial cells and lectins.

#### Bacterial adhesion to GCIY and acid tolerance of GCIY

The gastric cancer cell line GCIY was used to investigate microbe–host interactions. The adhesion assay was done as described previously (Shibahara-Sone et al., 2016) with minor modifications. Briefly, cells of WT or mutant #1476 were collected by centrifugation (3,000 *g*, 10 min, 4°C), washed once with Eagle's minimal essential medium (Nissui, Osaka, Japan) without antibiotics and containing 10% fetal bovine serum (Thermo Fisher Scientific), and resuspended in the same medium. GCIY cells were seeded into 96-well culture plates and grown to reach 2×10^3^ or 1×10^4^ cells/well. A sheet of GCIY cells (2×10^3^ or 1×10^4^ cells/well) was rinsed with fresh medium and incubated with the WT or #1476 suspension (1×10^5^, 1×10^6^, or 1×10^7^ CFU/well) or medium (negative control) at 37°C for 30 min in humidified air containing 5% CO_2_. The cell sheet was then rinsed in fresh medium three times, acidified fresh medium (pH 4.5) was added, and the cell sheet was incubated for 4.5 h. The acid-treated cell sheet was then rinsed in fresh medium three times, and cell morphology was observed by microscopy. The acid-treated cell sheet was then incubated in fresh (non-acidified) medium overnight, and viable cell counts and morphological changes were investigated.

### Method details

#### Proteinase K treatment or heat killing of BF-1

BF-1 cells were suspended in phosphate-buffered saline (PBS), and the optical density at 660 nm (OD_660 nm_) was adjusted to approximately 20. Proteinase K (200 μg/mL; GE Healthcare Life Sciences, Little Chalfont, England) was added, and the suspensions were incubated at 55°C for 1 h. For heat killing, cell suspensions prepared as above but without proteinase K were incubated at 95°C for 1 h. Treated cells were used in the mucin adhesion assay described below.

#### Construction of plasmids for transposon DNA

We constructed *E. coli*–*Bifidobacterium* shuttle vectors (pBDCNBb1F: chloramphenicol resistant; pBDSNBb1F: spectinomycin resistant) from pUC19 (Takara Bio), *Staphylococcus aureus* chloramphenicol-resistance gene or *Enterococcus faecalis* spectinomycin-resistance gene, and the replication origin of the *Bifidobacterium breve* plasmid pNBb1. The chloramphenicol-resistance gene was amplified from pBDCNBb1F by PCR (primer set: InFusion-FW2, ATATTGGCTCGAATTCGAAAAGGATTTTTCGCTACGCTCA; InFusion-RV2, AGTCGTTGGCAAGCTGATCTGGAGCTGTAATATAAAAACC). This fragment was introduced into an *Eco*RI–*Hin*dIII-digested EZ-Tn5 pMOD-2 Transposon Construction Vector (Epicentre, Madison, WI, USA) by using an In-Fusion HD Cloning Kit (Takara Bio). We named this construct pMOD-pBDcat. The chloramphenicol-resistance gene with mosaic end sequences was amplified from pMOD-pBDcat by PCR (primer set: InFusion-FW, TCATTGAGATGTCGACATTCAGGCTGCGCAACTGT; InFusion-RV, TGATTACGCCAAGCTTGTCAGTGAGCGAGGAAGCG). This fragment was introduced into *Sal*I–*Hin*dIII-digested pBDSNBb1F using the In-Fusion HD Cloning Kit. We named this plasmid pBDMcat (Figure S2C).

To enhance expression of the chloramphenicol-resistance gene in BF-1, the elongation factor (*tuf*) promoter of *B. breve* was amplified from *B. breve* genomic DNA (primer set: InF-pMOD-Bbr-EF-FW, TATTGGCTCGAATTCCACGCGCCTCACGATGAAG; InF-pMOD-Bbr-EF-RV, TTTATTAAAGTTCATTACTTTTGTCCTCCTGGACGTCTC) and introduced into pBDMcat, which was amplified (inverse primer set: InFusion-pBDM-Fw, ATGAACTTTAATAAAATTGATTTAGACAATTGG; InFusion-pBDM-Rv, GAATTCGAGCCAATATGCGAGAAC) by using the In-Fusion HD Cloning Kit, so that the upstream region of the chloramphenicol-resistance gene was replaced with the strong *tuf* promoter. We named this plasmid pBDMcat4 (Figure S2C). The transposon DNA region of pBDMcat4 was sequenced.

#### Plasmid extraction from *Bifidobacterium bifidum* for transposome preparation

Plasmids were extracted from *B. bifidum* as described by Anderson and McKay (1983) with a minor modification. Cells were collected from 100 mL of culture by centrifugation at 4,400 *g* at 20°C for 5 min and washed with 25 mL of 0.2 M glycine buffer (pH 10). Cells were suspended in 18.95 mL of 6.7% sucrose, 50 mM Tris-HCl, and 1 mM EDTA (pH 8.0). Lysozyme solution (5 mL; 10 mg/mL lysozyme [Sigma-Aldrich], 0.05 mg/mL mutanolysin [Sigma-Aldrich], 25 mM Tris-HCl [pH 8.0]) was added to the suspension, and the mixture was incubated at 37°C for 30 min. After adding 500 μL of 10 mg/mL RNase A (Sigma-Aldrich), the cell suspension was incubated at room temperature for 2 min, and 2.41 mL of 250 mM EDTA–50 mM Tris-HCl (pH 8.0) and 1.38 mL of 10% Triton X-100 were added. The mixture was then alkalinized by adding 1.38 mL of 3 N NaOH dropwise with flicking, and mixed gently by inversion at room temperature for 5 min. The lysate was neutralized by adding 2.48 mL of 2 M Tris-HCl (pH 7.0), mixed gently by inversion at room temperature for 3 min, and underwent phenol–chloroform extraction and isopropanol precipitation. The precipitate was air-dried and dissolved in 1 mL of TE buffer (10 mM Tris-HCl, 1 mM EDTA, pH 8.0).

#### Transposome complex formation

To enhance transformation efficiency, pBDMcat4 was introduced into BF-1 to be modified with the inherent restriction-modification system of BF-1 cells. Then, pBDMcat4 was extracted from the BF-1 cells and digested with *Pvu*II. Transposon DNA was purified with a QIAquick Gel Extraction Kit (Qiagen, Heidelberg, Germany). EZ-Tn5 Transposome was prepared by mixing 1 μL of transposon DNA (200–300 ng), 1 μL of 100% glycerol, and 2 μL of EZ-Tn5 Transposase (Epicentre). The EZ-Tn5 Transposome was stored at −30°C until use.

#### Transformation of *Bifidobacterium bifidum* BF-1

An overnight culture of BF-1 cells in MRS medium supplemented with 0.05% L-cysteine HCl was inoculated at a rate of 5% (v/v) into fresh MRS medium supplemented with 0.05% L-cysteine HCl and 0.2 M sucrose. Cells were grown at 37°C for about 4 h until reaching OD_660 nm_ = 0.7. Cells were harvested by centrifugation at 4,400 *g* at 0°C for 10 min and washed three times with ice-cold 1 mM Tris-HCl buffer (pH 9.0) containing 0.5 M sucrose. Cells were suspended in the same buffer to make a suspension of OD_660 nm_ = 50. Electroporation was done with 39 μL of electrocompetent cells and 1 μL of plasmid (100–200 ng) or transposome (50–75 ng DNA) in a 1-mm-path cuvette at 11.5 kV/cm, 200 Ω, 25 μF with a Gene Pulser II Electroporation System (Bio-Rad Laboratories, Hercules, CA, USA). Cells were transferred to 4 mL of MRS medium supplemented with 0.05% L-cysteine HCl and incubated anaerobically at 37°C for 3 h. Cells were harvested by centrifugation at 4,400 *g* at 20°C for 5 min, and the supernatant was discarded. The cells were resuspended in remaining medium and spread on an agar plate of MRS medium supplemented with 0.05% L-cysteine HCl containing 8 μg/mL of chloramphenicol, and the plate was incubated anaerobically at 37°C for 5 days.

#### Identification of transposon insertion sites

The transposon insertion sites were determined by direct sequencing of genomic DNA using a BigDye Terminator v3.1 Cycle Sequencing Kit (Thermo Fisher Scientific, Waltham, MA, USA). The mixture (10 μL) contained 1 μg of genomic DNA, 1.28 pmol of primer (DSq-Cm-Fw, TATTCAGGAATTGTCAGATAGGCCTAATGA or DSq-Cm-Rv, CCTGGACGTCTCGTGAGTTTCCTGCACCCT), 2 μL of Terminator Ready Reaction Mix, and 1 μL of 5× Sequencing Buffer. Cycle sequencing was done according to manufacturer's instructions. The reaction mixture for cycle sequencing was purified using XTerminator solution (Thermo Fisher Scientific) and SAM solution (Thermo Fisher Scientific). The samples were sequenced using a 3500xl Genetic Analyzer (Thermo Fisher Scientific).

#### Southern hybridization

Probe DNA was prepared by PCR using pMOD-pBDcat4 (pMOD-pBDcat with the *tuf* promoter of *B. breve* upstream of the chloramphenicol-resistance gene) as a template and primers Probe-Fw (TTCTCGGGTGTTCTCGCATAT) and Probe-Rv (GTTGGCTAGTGCGTAGTCGTT). Genomic DNA (2 μg) was digested with *Sal*I and underwent agarose gel electrophoresis. Probe DNA was labeled with AlkPhos Direct Labelling Module (GE Healthcare Life Sciences). The signal was detected by using CDP-Star Detection reagent (GE Healthcare Life Sciences) and LAS-3000 Imaging System (GE Healthcare Life Sciences). Labelling, blotting, hybridization, and detection of the signal were performed in accordance with the AlkPhos Direct Protocol (GE Healthcare Life Sciences).

#### SDS-PAGE of bacterial membrane proteins

We used the method reported by Gatti et al. (2001); in the case of bifidobacteria, cell surface proteins not anchored to cell walls were extracted. We assumed that precursors of sortase-dependent proteins (SDPs) would be associated with cell membranes. Briefly, a pellet of wet cells (approx. 50 mg) was suspended in 1 mL of solution 1 (0.05 M Tris-HCl [pH 7.5], containing 0.1 M CaCl_2_) and centrifuged (5,000 *g,* 10 min). This washing step was repeated twice, and the cells were finally washed in 1 mL of distilled water. The pellet was resuspended in 200 μL of solution 2 (0.01 M Tris-HCl [pH 8.0], containing 0.01 M EDTA, 0.01 M NaCl, and 2% SDS), incubated at room temperature for 1 h, and heated at 100°C for 5 min. The sample was allowed to cool to room temperature for 1 h and was then cooled to 4°C over 2 h. The samples were centrifuged (11,600 *g*, 10 min, 4°C), and the supernatants underwent SDS-PAGE.

#### Protein identification

Proteins were digested in the gel as previously described with minor modifications (Ishikawa et al., 2005). The target protein was stained with Quick CBB (Fujifilm Wako Chemical, Tokyo, Japan), cut from the gel, de-stained in 50% (vol/vol) acetonitrile in water, dehydrated in 100% acetonitrile, and dried in a vacuum desiccator. ProteoExtract All-in-One Trypsin Digestion Kit (Merck-Millipore, Burlington, MA, USA) was used for in-gel tryptic digestion. The peptide mixtures were applied to a liquid chromatography–quadrupole time-of-flight mass spectrometry system (QSTAR Elite; AB Sciex, Framingham, MA, USA) equipped with a Cadenza CD-C18 2.0×150 mm column (Imtakt, Kyoto, Japan) and eluted in 75 min at a flow rate of 0.2 mL/min with a 3%–40% linear gradient of acetonitrile containing 0.1% formic acid. The data of peptide mass fingerprinting were searched against the protein database generated from the BF-1 genome.

#### DNA and RNA extraction

Bacterial cells were harvested in the exponential growth phase and DNA was extracted with an Illustra Bacteria Genomicprep Mini Spin Kit (GE Healthcare Life Sciences).

A volume of bacterial culture in exponential growth phase was mixed with twice the volume of RNA Protect Bacteria Reagent (Qiagen) and centrifuged (20,000 *g*, 5 min, 4°C). The bacterial pellet was suspended in 1.0 mL of 50 mM Tris, 10 mM EDTA (pH 8.0), 10% (w/v) sucrose. Lysozyme (50 mg/mL, 50 μL; Sigma-Aldrich) was added. The suspension was incubated for 10 min at 37°C and centrifuged (20,000 *g*, 5 min, 4°C). RNA was extracted from the precipitated spheroplasts with RiboPure-Bacteria (Thermo Fisher Scientific). RNA integrity and concentration were checked by a Bioanalyzer 2100 (Agilent Technologies, Palo Alto, CA, USA) and RNA 6000 Series II Nano kit (Agilent Technologies).

#### Genetic complementation

The *E. coli–Bifidobacterium* shuttle vector pBDSNBb1F was used for genetic complementation. The *tuf* promoter of *B. breve* was amplified with the primer set ACGGCCAGTGAATTCCACGCGCCTCACGATGAAG and TGCTTCATATGTACTTTTGTCCTCCTGGACGTCTC, and BF1_0427 encoding the housekeeping sortase was amplified with the primer set GACTGACTCATATGAAGCATTCGCGCAACTCT and GATTACGCCAAGCTTCCCATTATCGCCGCCAACCG. The *tuf* promoter amplicon was digested with *EcoR*I and *Nde*I, and the BF1_0427 amplicon was digested with *Nde*I and *Hind*III. The digested fragments were subcloned into the multi-cloning site of pBDSNBb1 digested with *EcoR*I and *Hind*III. The sequences of plasmid constructed for BF1_0427 complementation was confirmed by capillary sequencing with a 3500xl Genetic Analyzer (Thermo Fisher Scientific). The construct was introduced into the non-adhesive mutant #1476 by electroporation, and spectinomycin-resistant colonies were isolated on MRS plates containing spectinomycin (375 μg/mL).

#### SPR assay

Recombinant SDPs were prepared using the pCold system (Takara Bio) and a Ni-NTA Spin Kit (Qiagen) according to the manufacturers' protocols. The primers were designed to produce mature SDPs without the N-terminal signal peptide and C-terminal membrane-spanning region (Table S1). A Biacore T200 instrument (GE Healthcare Life Sciences) was used for the SPR assay according to the manufacturer's instructions. The sensor chip was CM5 and the buffer was HBS-P. Mucin described above (Sigma-Aldrich) was immobilized onto the chip; lectins WGA and SBA (Vector Labs) were used as positive controls and BSA (Sigma-Aldrich) as a negative control for binding to immobilized mucin. Each protein was analyzed twice on different chips.

#### Microarray analysis

A custom microarray (Agilent Technologies) based on the genome sequence of BF-1 was used for microarray analysis. According to the manufacturer's protocol, the microarray images were scanned by a SureScan microarray scanner (Agilent Technologies) and analyzed by Feature Extraction 12.0 (Agilent Technologies). Microarray data were analyzed by GeneSpring 14 (Agilent Technologies). The array format and data were submitted to the Gene Expression Omnibus (GEO: GSE175843). Because bacterial transcriptomes are reproducible, no replications were performed; instead, we examined all three mutants to discover common gene expression profiles. The expression of genes shown in Figure 5 was verified by RT-qPCR.

### Quantification and statistical analysis

Statistical significance was determined with Welch *t* test for two groups and one-way ANOVA with post hoc Tukey honestly significant difference (HSD) test for more than two groups in R software (https://www.R-project.org/). Differences were considered statistically significant at p values of ≤0.05.

## Data Availability

Complete genome sequence and microarray data have been deposited and are publicly available as of the date of publication. Accession numbers are listed in the key resources table. This study did not generate any computational codes. Any additional information required to reanalyze the data reported in this paper is available from the lead contact upon request.
