# Supplementary material for: Critical roles of a housekeeping sortase of probiotic Bifidobacterium bifidum in bacterium–host cell crosstalk
Source: iScience. 2021 Oct 28;24(11):103363. doi: 10.1016/j.isci.2021.103363 (PMC8603203; doi:10.1016/j.isci.2021.103363)
Supplement: Document S1. Figures S1–S6 and Table S1 [file mmc1.pdf]

iScience, Volume *24*

Supplemental information

Critical roles of a housekeeping sortase of probiotic *Bifidobacterium bifidum* in bacterium–host cell crosstalk

Eiji Ishikawa, Tetsuya Yamada, Kazuaki Yamaji, Masaki Serata, Daichi Fujii, Yoshinori Umesaki, Hirokazu Tsuji, Koji Nomoto, Masahiro Ito, Nobuhiko Okada, Masato Nagaoka, and Atsushi Gomi

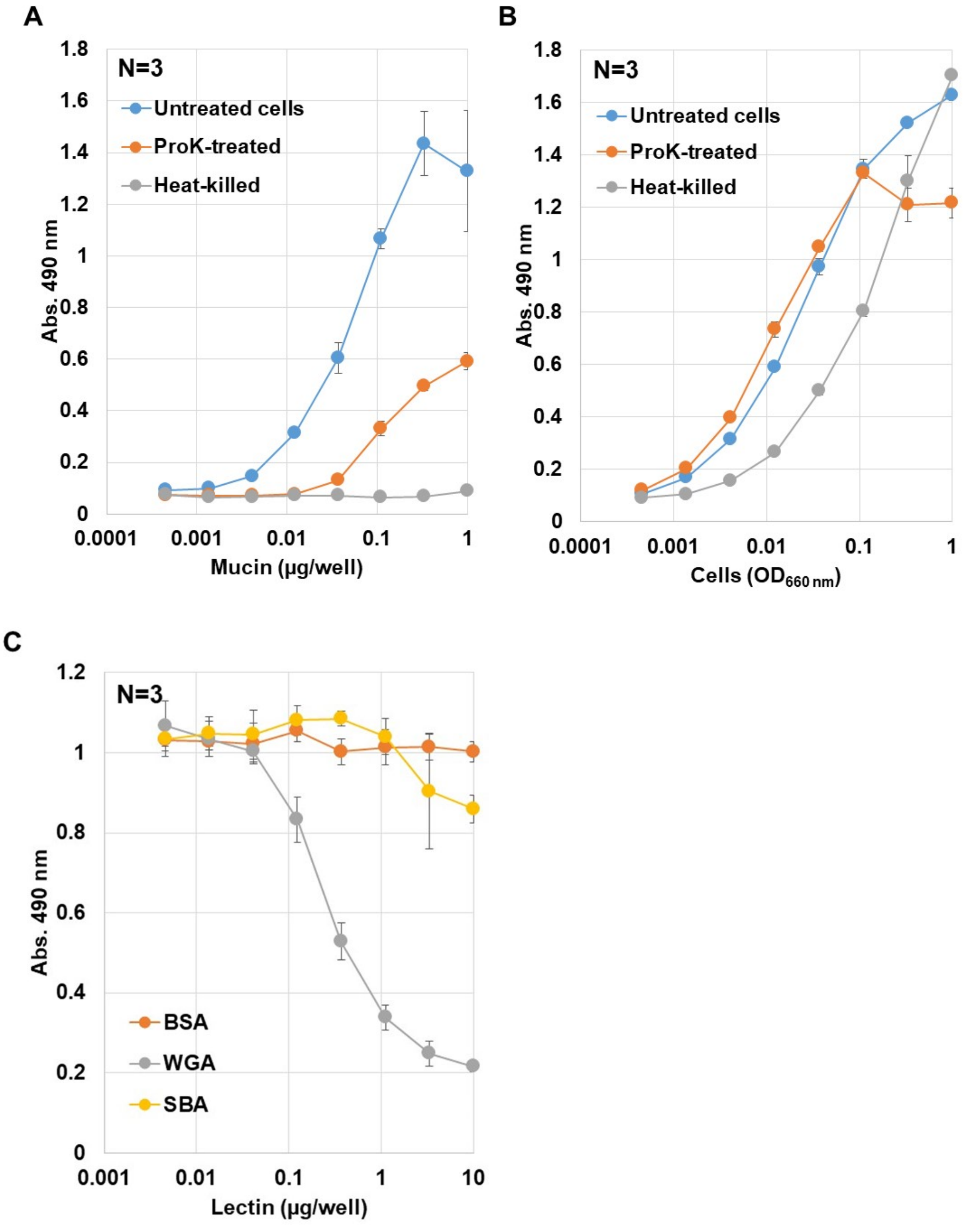

Figure S1. Effects of proteinase K treatment and heat treatment on adhesion of *Bifidobacterium bifidum* YIT 10347 (BF-1) to mucin. Related to STAR methods.

(A) Mucin adhesion. Bacteria adhering to mucin-coated plates were detected by ELISA.

(B) Antibody reactivity. Serial dilution of bacteria immobilized onto plates were detected by ELISA.

Data shown in (A) and (B) are means of 3 replicates ± SD.

Denaturation of cell surface proteins abolished mucin adhesion, whereas antibody reactivity was almost the same among samples.

(C) Effects of lectins on adhesion of BF-1 to mucin. Wheat germ agglutinin (WGA) strongly inhibited adhesion, whereas soy bean agglutinin (SBA) only slightly inhibited adhesion. Bovine serum albumin (BSA; negative control) had no effect. Other lectins (e.g., concanavalin A) also had no effect (data not shown).

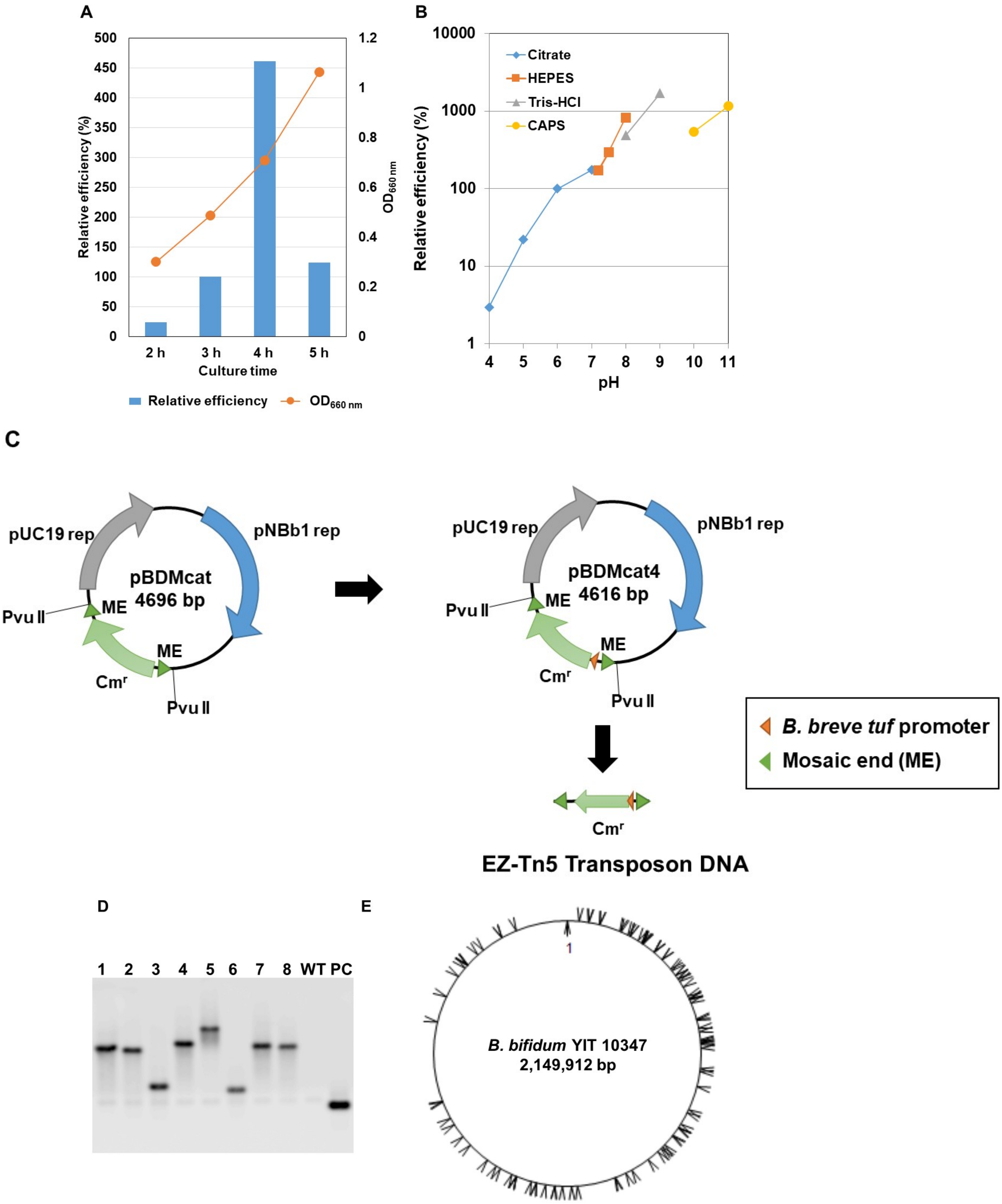

Figure S2. Transposon system developed for BF-1. Related to Table 1.

(A) Optimization of growth phase for preparing electrocompetent cells.

(B) Optimization of cell suspension buffer and pH for preparing electrocompetent cells.

(C) Preparation of the transposon DNA fragment. pBDMcat4 was constructed as described in METHODS DETAILS by replacing the promoter of the chloramphenicol-resistance gene Cm^r with the *tuf* promoter of *Bifidobacterium breve* (orange arrowhead). The EZ-Tn5 transposon DNA was cut out of pBDMcat4.

(D) Confirmation of random single insertions by Southern hybridization. Chromosome DNA of the wild type (WT) and 8 clones selected randomly from the library was digested with *Sal*I. Transposon DNA containing the chloramphenicol-resistance gene was used as a positive control (PC).

(E) Positions of transposon insertion sites identified in 100 clones in the BF-1 genome. Transposons were inserted evenly throughout the genome.

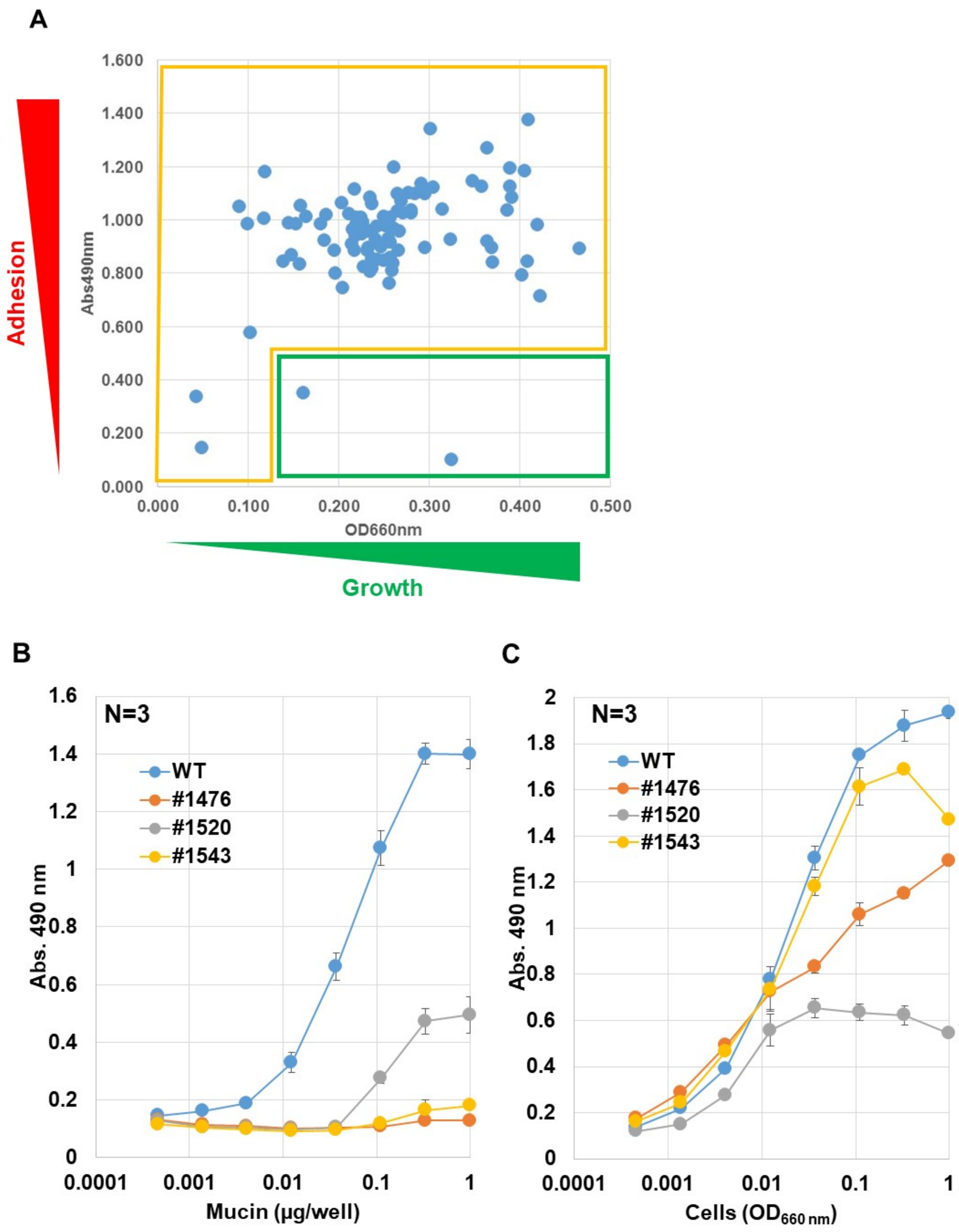

Figure S3. Non-adhesive mutants from the Tn-mutagenesis-derived library identified by ELISA-based adhesion assay. Related to Table 1.

(A) Screening for non-adhesive mutants. Optical density of an overnight culture at 660 nm (*x*-axis) was plotted against absorbance at 490 nm in ELISA-based mucin adhesion assay (*y*-axis). Non-adhesive mutants with good growth inside the green box were selected.

(B, C) Mucin adhesion (B) and antibody reactivity (C) of the three selected mutants. Mutants #1476 and #1543 were non-adhesive mutants with normal antibody reactivity, whereas #1520 was a mutant defective in antibody reactivity.

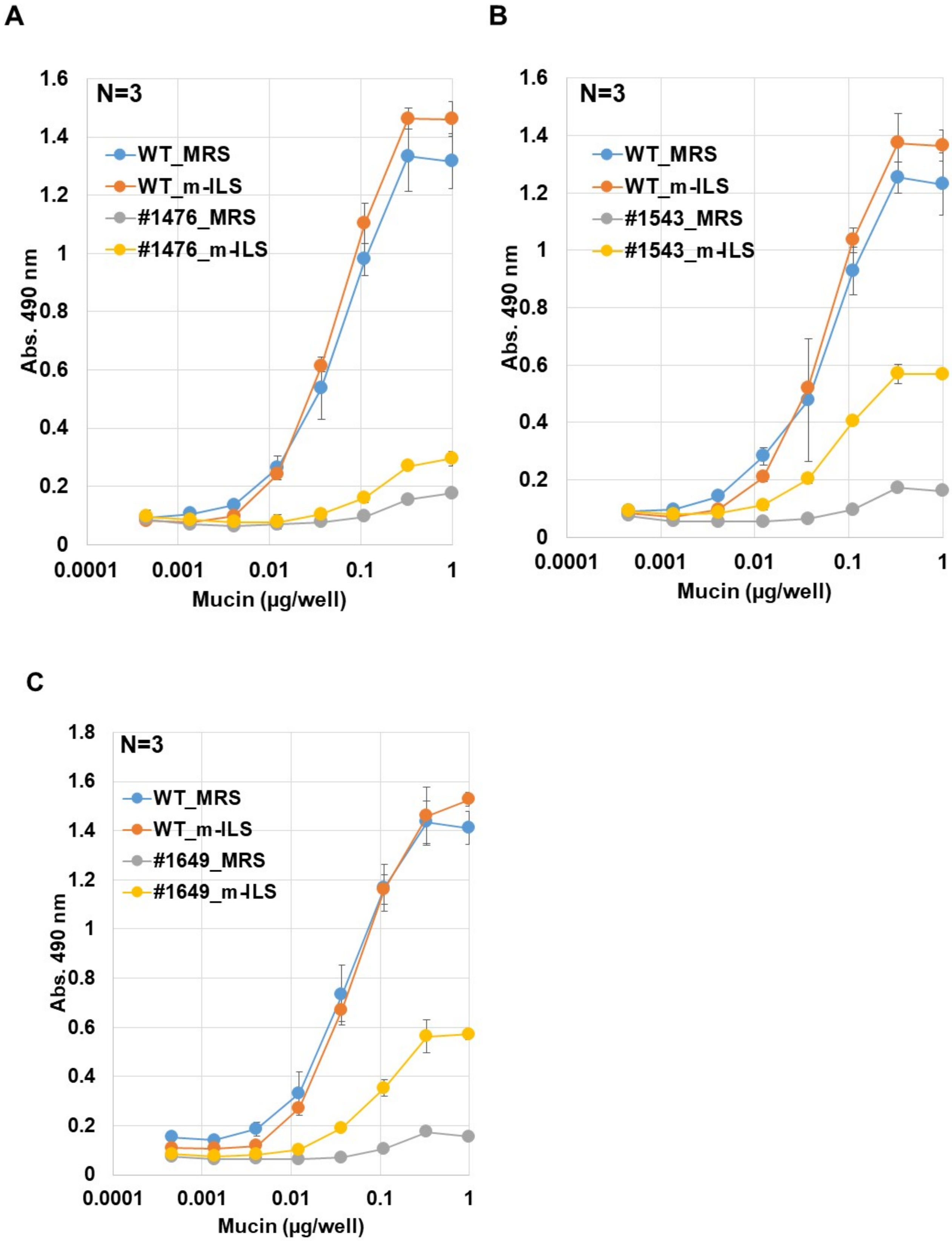

Figure S4. Mucin adhesion of mutants in MRS and m-ILS culture media. Related to Fig. 2.

Mucin adhesion of partially recovered in when cultured in m-ILS in comparison with MRS, whereas mucin adhesion of #1476 did not recover.

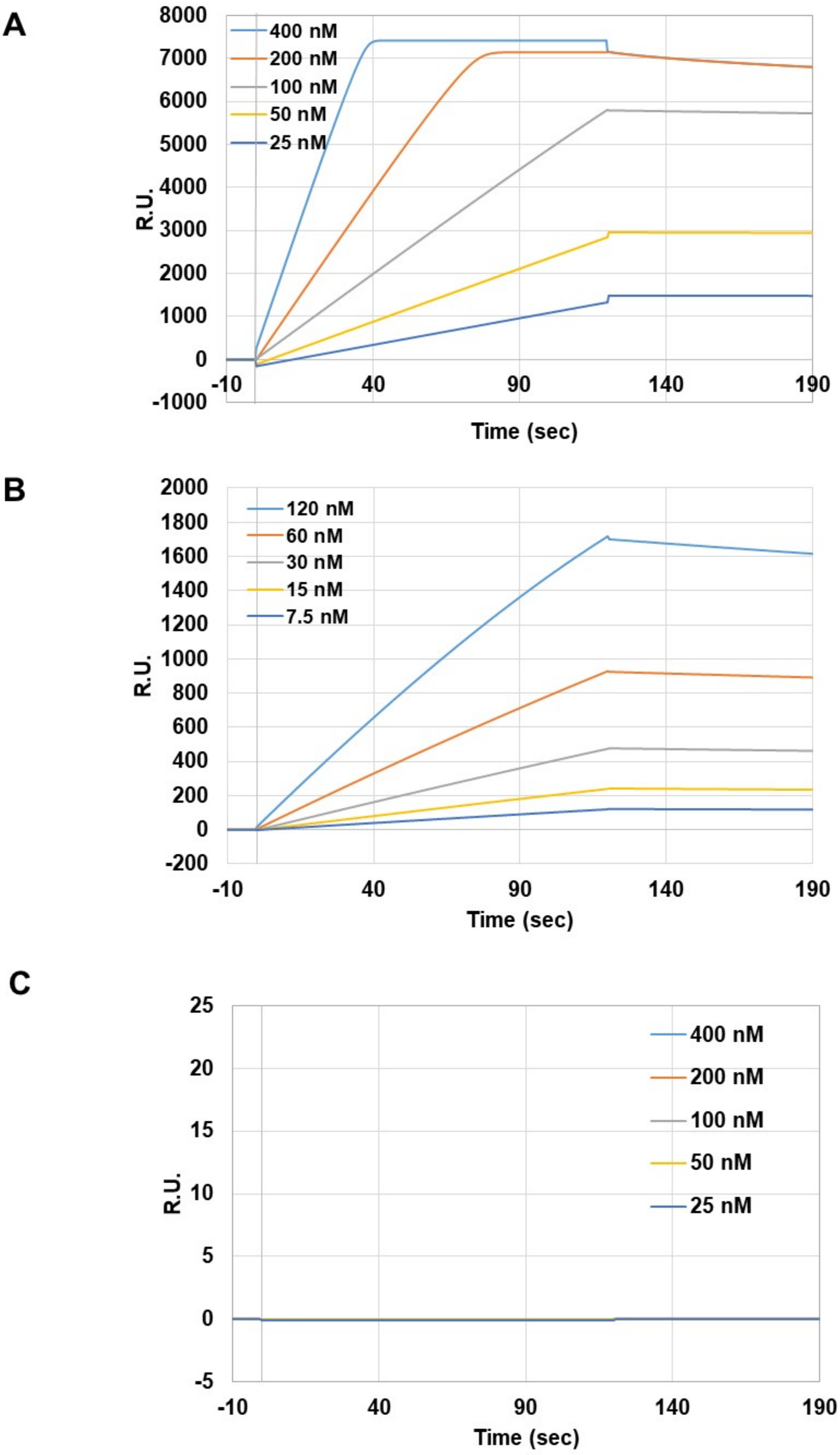

Figure S5. Surface plasmon resonance (SPR) assay for mucin adhesion. Related to Fig.4.

Wheat germ agglutinin (A) and soy bean agglutinin (B) were used as positive controls, and bovine serum albumin (C) was included as a negative control. The lectins showed concentration-dependent responses.

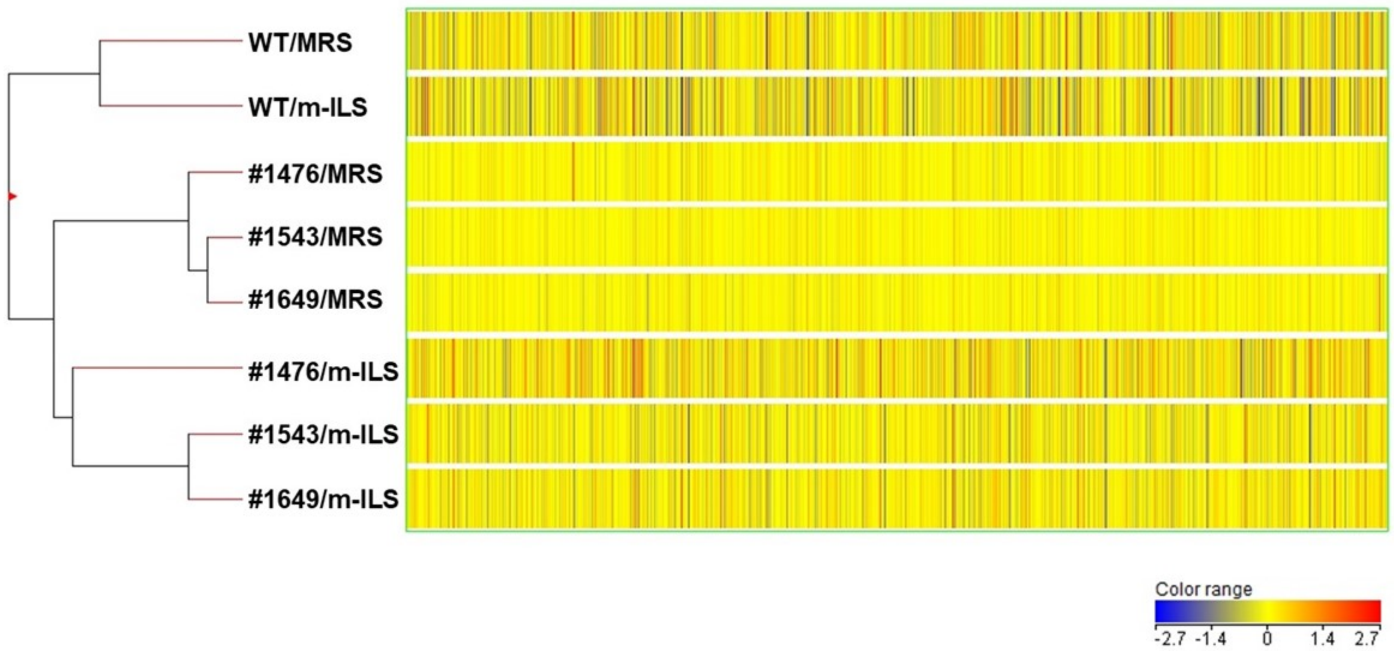

Figure S6. Effects of housekeeping sortase deficiency and media on BF-1 transcriptomes. Related to Fig. 5

Impacts of media (MRS vs. m-ILS) were greater in non-adhesive mutants than in WT. Expression profile of #1476 differed from those of #1543 and #1649, consistent with the difference in adhesion properties shown in Figure S4.

Table S1. Primers used for preparing recombinant SDPs. Related to STAR Methods.

ID	Predicted function	Forward (5′⇒3′)	Reverse (5′⇒3′)
BF1_0030	α-N-Acetylglucosaminidase	CGAGGGATCCGAATTCACACTCATGTGCGGCCTGA[1)]	CGACAAGCTTGAATTCCGTCTTCGAGAGGGTTTCC
BF1_0032	Metallophosphoesterase	CGAGGGATCCGAATTCGCCTACGCGCAGCCGACCG	CGACAAGCTTGAATTCGGTCTGCGACAGCTGCTCG
BF1_0041	β-N-Acetylglucosaminidase	CGAGGGATCCGAATTCGTGCAGACGGCGTACGCGG	CGACAAGCTTGAATTCGGTCTTGGAGATGTCGTCA
BF1_0056	β-N-Hexosaminidase	CGAGGGATCCGAATTCCCCGAGAAGGCCGTGGACG	CGACAAGCTTGAATTCGGTCTTGGCGACCTCGTCA
BF1_0115	Sialidase	CGAGGGATCCGAATTCGTCGAGCCCGTGCAGACCA	CGACAAGCTTGAATTCGGTGGCGCTCAGCCCGCCC
BF1_0155	β-N-Acetylglucosaminidase	CGAGGGATCCGAATTCGTTCCGGCCATGGCGGCCA	CGACAAGCTTGAATTCCGTGTTGGACAGCTTGCCG
BF1_0172	Hypothetical protein	CGAGGGATCCGAATTCGCGGTGACGTTGAGCAGGG	CGACAAGCTTGAATTCGGTATGAGACAGCACCGGC
BF1_0251	Minor extracellular serine protease	CGAGGGATCCGAATTCACGCCGACTCTGTCGACCG	CGACAAGCTTGAATTCCGTCTTGGCGACGCCGGCA
BF1_0298	Sialidase	CGAGGGATCCGAATTCGCGGACGAAACACCCCAAG	CGACAAGCTTGAATTCGGTTTTCGCCACAGCGTCC
BF1_0299	Exo-α-sialidase	CGAGGGATCCGAATTCAGCGATGATGCTGACATGC	CGACAAGCTTGAATTCGGTCTTGGACAGGCCGGGC
BF1_0365	α-L-Arabinofuranosidase	CGAGGGATCCGAATTCGCCCCGCCATTACTTGCAT	CGACAAGCTTGAATTCAGTATGCGACAACTTTGTT
BF1_0485	Sucrose symporter scrT	CGAGGGATCCGAATTCCGCCCCGGCATCGATGATC	CGACAAGCTTGAATTCGGTGACGGCCACACCGACC
BF1_0505	Bacillolysin /Chitinase	CGAGGGATCCGAATTCGCGGAACGCCTTGTGGGCG	CGACAAGCTTGAATTCGGTCTTCGACAGGCCGGGC
BF1_0510	α-Fucosidase	CGAGGGATCCGAATTCGTCGGCACGACGAGAGCGG	CGACAAGCTTGAATTCGGTCTTCGCGACACCGTCG
BF1_0575	Endo-α-N-acetylgalactosaminidase	CGAGGGATCCGAATTCAGCGAAGTCACTGATGTCT	CGACAAGCTTGAATTCCGTCTTGGAGATGGCGTTG
BF1_0619	Hypothetical protein	CGAGGGATCCGAATTCGCGGCGCTGCTCACCGCGG	CGACAAGCTTGAATTCGGTGCGGGAGAGCGGCTCG
BF1_0708	Autotransporter adhesin	CGAGGGATCCGAATTCATCTGGGTGAGCTCCATCA	CGACAAGCTTGAATTCGGTCACGCTGATGTACCAA
BF1_0740	Polysaccharide-degrading enzyme	CGAGGGATCCGAATTCGCCGATGCGACGCCAGCGG	CGACAAGCTTGAATTCGGTCTTGGACAGACCGTTG
BF1_0762	β-Galactosidase	CGAGGGATCCGAATTCGCCACAGTGGCCTTGTCAA	CGACAAGCTTGAATTCGGTCTTGCTCAGTGCGCTG
BF1_1178	β-N-Acetylhexosaminidase	CGAGGGATCCGAATTCCAGCACAACGCAGAGGCGG	CGACAAGCTTGAATTCGGTCTCGGCGACGACATCA
BF1_1357	Membrane-associated phospholipid phosphatase	CGAGGGATCCGAATTCGACACTCCCTCGTACGGCA	CGACAAGCTTGAATTCGGTCTTCGACAGCGGCTGC
BF1_1442	Lipoprotein	CGAGGGATCCGAATTCGAGGGTACGACTGATATTG	CGACAAGCTTGAATTCCGTGTGAGCCAGCCGGACG
BF1_1449	Glycosyl hydrolase	CGAGGGATCCGAATTCGCCGGAGGAGACGTCATCT	CGACAAGCTTGAATTCGGTCGCGGCGATGGACTGG
BF1_1506	α-1,3/4-Fucosidase	CGAGGGATCCGAATTCGCGAATCCGGCGGAATACC	CGACAAGCTTGAATTCGGTCTTTGCGATCACGTCA
BF1_1526	Cation-transporting ATPase, E1-E2 family	CGAGGGATCCGAATTCGCGATACTGCGCGAGAACA	CGACAAGCTTGAATTCGGTCATCGCGACCACCTCG
BF1_1579	Lacto-N-biosidase	CGAGGGATCCGAATTCCTGATGGTCGGTGGCATGT	CGACAAGCTTGAATTCGGTGGCGCTGAGCTTGCCG

[1)] Restriction enzyme recognition sites are underlined.
